# Headache management: pharmacological approaches

**DOI:** 10.1136/practneurol-2015-001167

**Published:** 2015-07-03

**Authors:** Alex J Sinclair, Aaron Sturrock, Brendan Davies, Manjit Matharu

**Affiliations:** 1 Department of Neurobiology, School of Clinical and Experimental Medicine, College of Medical and Dental Sciences, The University of Birmingham, Birmingham, UK; 2 Neurology Department, University Hospitals Birmingham NHS Trust, Queen Elizabeth Hospital Birmingham, Birmingham, UK; 3 Department of Neurology, Royal Stoke University Hospital, Stoke-on-Trent, UK; 4 Headache Group, Institute of Neurology, London, UK

**Keywords:** HEADACHE, MIGRAINE

## Abstract

Headache is one of the most common conditions presenting to the neurology clinic, yet a significant proportion of these patients are unsatisfied by their clinic experience. Headache can be extremely disabling; effective treatment is not only essential for patients but is rewarding for the physician. In this first of two parts review of headache, we provide an overview of headache management, emerging therapeutic strategies and an accessible interpretation of clinical guidelines to assist the busy neurologist.

## Background

Headache is listed among the WHO's major causes of disability with a global prevalence of 47% (symptoms occurring at least once in the past year). It is the commonest neurological syndrome presenting to primary care with 3% of adults consulting a general practitioner for headache each year.^1^ Women are disproportionately affected (3:1) and its higher prevalence among those of working age adds to the socioeconomic burden with loss of productivity, estimated at £2.25 billion per year in the UK.^2^

In neurological practice, headache accounts for 25% of new referrals and yet a large proportion of these patients feel dissatisfied even if they make it to the neurology clinic, partly due to a reported lack of interest in the disorder by the reviewing neurologist.^2^ Most patients with headache have migraine, and when this diagnosis is made it is usually correct (98%).^3^ However, a quarter of patients with migraine have as their diagnosis missed. Furthermore, of those identified as a non-migrainous primary headache, 82% actually have migraine or probable migraine.^3^

Neurologists consequently see a lot of patients with headache and the condition is very disabling for individuals. A diagnosis, empathy and effective treatment make a huge difference to the patient and can be very rewarding for the clinician. The purpose of this article, the first of two linked articles, is to provide an up-to-date overview of the pharmacological management of common headache disorders (as well as a limited number of non-pharmaceutical strategies).

## The common primary headache disorders

In European populations, the annual sex-adjusted prevalence for tension-type headache is 35%, for migraine is 38%, but for cluster headache is only 0.15%.^4^ ^5^ These three together comprise the most prevalent primary headache disorders. The remaining primary headache diagnoses—all relatively rare—include paroxysmal hemicrania, hemicrania continua, short-lasting unilateral neuralgiform headache attacks, hypnic headache and new daily persistent headache. Table 1 outlines the features that help to distinguish these disorders.

**Table 1:** Key clinical features that assist the differentiation of more common headache disorders

Headache	Tension-type headache	Migraine	Trigeminal autonomic cephalalgias				Trigeminal neuralgia
			Cluster headache	Paroxysmal hemicrania	Hemicrania continua	SUNCT/SUNA	
Sex (M:F)	4:5	3:1	5:1	1:1	1:2	3:2	2:3
Duration	30 min to 7 days (episodic)	4–72 h	15–180 min	2–30 min	Continuous headache	1–600 s	1–120 s
Frequency	Episodic or chronic (variable from rare to daily)	Episodic or chronic (variable from rare to daily)	1–8/day	>5 daily for more than half of the time	Continuous headache	>1 daily for more than half of the time	Very variable frequency
Pain type							
Location	Bilateral	Unilateral or bilateral	Unilateral	Unilateral	Unilateral	Unilateral; V1/V2>V3	Unilateral; V2/V3>V1
Quality	Pressing/tightening (non-throbbing)	Throbbing	Variable	Variable	Variable	Neuralgiform pain	Neuralgiform pain
Severity	Mild to moderate	Moderate to severe	Very severe	Very severe	Moderate to very severe	Very severe	Very severe
Migrainous symptoms	–	+++	+/–	–	+/–	–	–
Autonomic features	No	+/–	+++	+++	+++	+++	Sparse
Triggers			Alcohol (within 30 min)			Cutaneous	Cutaneous
Indometacin response	+/– (as simple analgesic)	± (as simple analgesic)	–	+++	+++	–	–

Based on framework of International Headache Society Classification Criteria.

Chronic migraine is defined as headache occurring on 15 or more days per month for more than 3 months, which has the features of migraine headache on at least 8 days per month. Chronic cluster headache and paroxysmal hemicrania are defined as headache attacks occurring for more than 1 year without remission or with remission periods lasting less than 1 month.

SUNA, short-lasting unilateral neuralgiform headache with cranial autonomic symptoms; SUNCT, short-lasting unilateral neuralgiform headache attacks with conjunctival injection and tearing.

Migraine is a common and disabling primary headache disorder and is the focus of this article. We also briefly consider the other common primary headache disorders, tension-type headache, cluster headache and also the management of medication-overuse headache.

## Migraine

### The changing theories of migraine

The *vascular hypothesis* was central to early understanding of migraine pathophysiology. Its central premise was that intracranial vasoconstriction caused the aura, while reflexive secondary vasodilatation generated pain through perivascular nerves. However, advances in intracerebral blood flow imaging have largely refuted this hypothesis.

The *neurovascular hypothesis* posits that migraine is a disorder of the endogenous pain modulating systems, particularly the subcortical structures. These include diencephalic and brainstem nuclei that can modulate the perception of activation of the trigeminovascular system, which carries sensory information from the cranial vasculature to the brain. Moreover, the involvement of a multisensory disturbance that includes light, sound and smell, as well as nausea, suggests the problem may more broadly involve central modulation of afferent traffic. Brain imaging studies in migraine suggest that subcortical structures are important causing migraine. Thus, it may be considered an inherited dysfunction of sensory modulatory networks with the dominant disturbance affecting abnormal processing of essentially normal neural traffic.

### Diagnosis and assessment

The first step is the accurate and positive diagnosis of migraine; this is usually straightforward. To select the most appropriate management, the migraine phenotype needs to be categorised as either episodic (occurring on fewer than 15 days a month) or chronic (occurring on more than 15 days a month, for over 3 months and with migrainous headaches on at least 8 days a month). The clinician should scrutinise the history for sinister features. If there is a *strictly unilateral* headache, consider hemicrania continua and conduct an indometacin challenge. Look out for medication-overuse headache, as this frequently coexists with migraine.

We frequently use a headache diary to quantify the headache and to provide a contemporary record of pain, as otherwise this can suffer from recall bias. The diaries also assist when monitoring the treatment response, identifying triggers, the relationship to menses and the frequency of analgesic use. Questionnaires can help to determine the degree of disability at baseline and to evaluate the response to treatment (such as the Headache Impact Test (HIT 6), the Migraine Disabilities Assessment Test (MIDAS)). The Hospital Anxiety and Depression Scale (HADS) can also help to assess the emotional and psychiatric comorbidity. This is particularly important in these patients and may influence the decision on preventative therapy, since many of these can exacerbate depression and anxiety.

### Non-pharmacological management of migraine

Although an enquiry about migraine triggers is useful, in reality certain triggers cannot be avoided (eg, lack of sleep, too much sleep, stress, heat). It may also be difficult to distinguish a true migraine trigger from premonitory symptoms occurring in the period before an attack (food cravings, fatigue, irritability).

Patients who seek a therapeutic intervention yet are reluctant to use medication have several options. Neurostimulation is one possibility and we shall cover this in the linked article. The UK's National Institute of Health and Care Excellence (NICE guideline CG 150) also advocates acupuncture an alternative second-line therapy for migraine. Although there are limited data to support its use in migraines, it is safe, and many patients have at least considered this before attending the clinic. A large study comparing acupuncture with a sham procedure and with standard migraine prophylactics found no significant difference between treatment groups in the number of migraine days, although all interventions were effective.^6^ Furthermore, acupuncture appears to improve health-related quality of life for many chronic migraineurs at relatively low cost.^7^ In many hospitals, the physiotherapy department offers an acupuncture service, but patients can also access it from primary care.

Cognitive behavioural therapy with a trained clinical psychologist may help, particularly combined with medication, but there is very limited evidence of efficacy. Many patients understandably have negative thoughts and emotions linked to the headache pain. Cognitive behavioural therapy can help to explore these and potentially lessen this burden, thereby also reducing anxiety.

### Acute management of migraine

Practice has gradually shifted from a treatment paradigm involving gradually escalating abortive therapy towards now starting with combination therapy—non-steroidal anti-inflammatory drugs (NSAIDs) or paracetamol and triptan—from the outset (box 1).^8^ This shift follows the NICE (CG150) guideline's cost–utility analysis, which evaluated costs and Quality-Adjusted Life Years (QALY) outcome measures. Based on this analysis, triptan plus NSAIDs combination was more effective than other acute migraine approaches, including triptan monotherapy (box 1 shows costs for various triptans); this, therefore, offers patients the best hope of rapid migraine resolution for an acute attack.^9^ The guideline's recommendation includes an antiemetic, even if nausea is not pronounced, to counter gastric stasis and so facilitating tablet absorption and pain relief. Last year, the European Medicines Agency issued a warning about domperidone being associated with a small risk of sudden cardiac death and fatal arrhythmias, particularly in those aged over 60 years. They recommended avoiding its prolonged use or doses above 30 mg daily. Patients taking medications that prolong the electrocardiography Q wave to T wave interval should avoid it and those with cardiac comorbidities should exercise caution. NICE recommends avoiding ergots and opioids.

Box 1 **Acute management of migraine**

Acute migraine management

National Institute of Health and Care Excellence (NICE) guidelines

1. Combination therapy: triptan+non-steroidal anti-inflammatory drug (NSAID) or paracetamol+antiemetic
2. Alternatively (per patient request):
  - a single agent (triptan, NSAID or paracetamol)±antiemetic

NSAID

- Aspirin 600–900 mg (ideally effervescent)
- Ibuprofen 600–800 mg
- Naproxen 500–1000 mg
- Diclofenac 50–75 mg (or 100 mg suppository)
- Tolfenamic acid 200 mg

Antiemetics

For nausea and/or as a prokinetics such as

- Domperidone 10 mg up to three times a day (or 60 mg suppository)
- Metoclopramide 10 mg
- Prochlorperazine 3–6 mg as buccal preparation

### The triptans

We advise patients to use a triptan at the start of the headache phase of a migraine attack, since there is no evidence of efficacy if taken during preceding aura.^10^ Using a triptan at the start of the headache may reduce headache recurrence, prevent disability and possibly reduce central sensitisation. The difficulty arises in chronic migraineurs, since taking the triptan early can lead to a pattern of increasing use and medication-overuse headache. We try to help these people to identify early features that might indicate an ensuing severe migraine attack, as the triptan should be reserved for these headaches. It is important to explain that the triptan should be used, on average, on no more than 2 days per week (10 days per month) to reduce the risk of a triptan-overuse headache.

**Figure 1:**
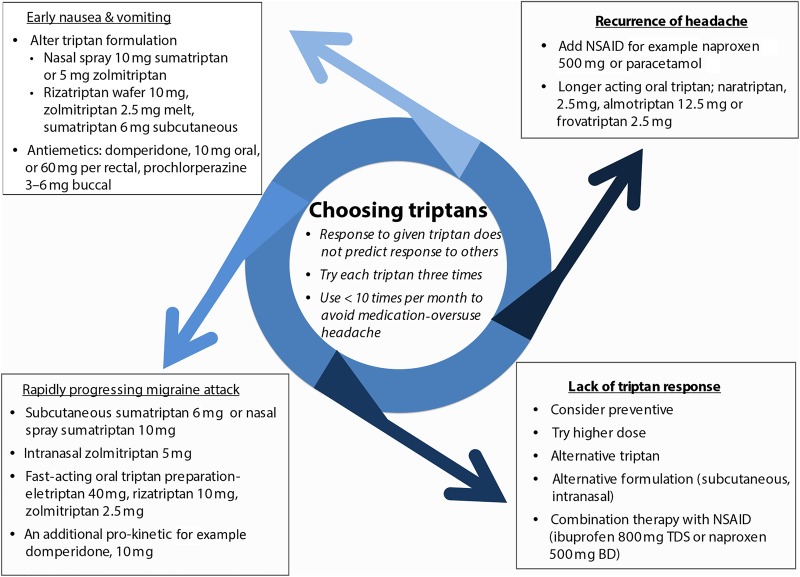
Triptan selection in acute migraine. An approach to triptan selection based on the characteristic of acute attacks. BD, twice daily; NSAID, non-steroidal anti-inflammatory drug; TDS, three times a day.

Our own practice is to try any particular triptan on at least three occasions before assessing its efficacy. Poor efficacy or tolerability with a single triptan does not reliably predict response to another, so it is definitely worthwhile working through different agents. However, choosing the right triptan for a given situation can be difficult, especially when considering second-line or third-line choices. Figure 1 and the following section may assist this decision.

#### Which triptan and how do they work?

The seven triptans—almotriptan, eletriptan, frovatriptan, naratriptan, rizatriptan, sumatriptan, zolmitriptan—are 5HT_1B/1D_ receptor agonists with vasoconstrictive effects on blood vessels. The choice of triptan depends on efficacy, side effects, the duration of the headache, coexisting vomiting and cost (table 2).

**Table 2:** The triptans

Triptan	Peak serum concentration	Half-life	Usual dose (maximum daily dose)	Cost (per tablet)
Almotriptan	1.5–2 h	3.5 h	12.5 mg (25 mg)	3.0 GBP (12.5 mg)
Eletriptan	1.5–2 h	4 h	40 mg (80 mg)	3.8 GBP (40 mg)
Frovatriptan	2–4 h	26 h	2.5 mg (5 mg)	2.8 GBP (2.5 mg)
Naratriptan	2–3 h	6 h	2.5 mg (5 mg)	3.8 GBP (2.5 mg)
Rizatriptan	1–1.5 h	2 h	10 mg (20 mg)—same recommendation for rizatriptan melt	4.5 GBP (5 mg)
Sumatriptan	2–3 h	2 h	50–100 mg (300 mg)	0.3 GBP (50 mg)
Sumatriptan subcutaneous	12 min	1.9 h	6 mg (12 mg)	21.2 GBP (per injection)
Sumatriptan intranasal	1–1.5 h	2 h	10–20 mg (40 mg)	5.9 GBP (per dose)
Zolmitriptan	1–1.5 h	2.5 h	2.5–5 mg (10 mg)	3.8 GBP (2.5 mg)
Zolmitriptan intranasal	15 min	3 h	5 mg into one nostril, once (10 mg)	11.0 GBP (per spray)

Drugs listed alphabetically. All doses apply to the oral route, except where otherwise specified.

GBP, pounds sterling.

#### Efficacy of triptans

Efficacy data at 2 h favour eletriptan, then rizatriptan, followed by zolmitriptan over sumatriptan, almotriptan, naratriptan, and frovatriptan. At 24 h, eletriptan performed best, followed by zolmitriptan and then almotriptan, rizatriptan and sumatriptan (100 mg).^11^

However, the most effective use of a triptan depends partly upon its route of administration. Thus, the most effective sumatriptan preparation for acute relief at 2 h (based on numbers needed to treat) was subcutaneous 6 mg, then intranasal 20 mg and then oral 100 mg formulations.^12^ While subcutaneous sumatriptan is most effective, it has more adverse events and costs more than triptans by other routes. The route of administration of the triptan is therefore best tailored to the individual's migraine attack. For example, if the migraine has a rapid onset, patients may try a fast-acting oral triptan such as rizatriptan, zolmitriptan or eletriptan, or nasal preparations that are faster still (zolmitriptan and sumatriptan); alternatively, the subcutaneous sumatriptan, which has the fastest onset of action. Other migraine features necessitate specific strategies (as outlined in figure 1).

#### Safety and side effects

We find the triptans to be well tolerated overall. The reported incidence of minor adverse effects does not differ markedly between the triptans, but depends on speed of onset of action; people taking subcutaneous sumatriptan report more adverse effects than those taking oral sumatriptan. The triptans with a longer half-life and slower onset of action, such as naratriptan and frovatriptan, have fewer side effects. The adverse effects of the oral triptans are similar, although dizziness and sedation occur more with rizatriptan and zolmitriptan than with sumatriptan and naratriptan.^13^

There are cardiovascular safety concerns associated with triptan use due to the presence of 5HT_1B_ receptors on vascular smooth muscle. We avoid triptans in people with uncontrolled hypertension, cardiovascular and/or cerebrovascular disease. However, in clinical trials, cardiovascular complications were fewer than one per million exposed, and a recent systematic review of cardiovascular safety data identified no strong cardiovascular safety issues.^14^ Triptan sensations such as burning or tingling in the chest or limbs are relatively common (7%), but clinicians can reassure patients that this is not associated with cardiac ischaemia.^15^

In 2006, the US Food and Drug Administration (FDA) identified the potential for developing a serotonin syndrome when taking triptans together with selective serotonin reuptake inhibitors (SSRIs) and serotonin/norepinephrine reuptake inhibitors (SNRIs).^16^ However, on further evaluation only 10 of the original 36 cases identified were actually serotonin syndrome and it was concluded that triptan use did not need to be restricted in patients on SSRIs or SNRIs.^17^ We use triptans in these patients where indicated but counsel patients to be aware of the features of serotonin syndrome (agitation, nausea, palpitation and sweating). Triptans should not be co-prescribed with ergotamine (within 24 h) or monoamine oxidase inhibitors (within 2 weeks).

#### Triptans in pregnancy and breast feeding?

The major concern for migraine medications during pregnancy is of potential teratogenicity; our practice during pregnancy is to continue triptans only if benefit outweighs risk. The sumatriptan pregnancy register (626 pregnancies exposed to sumatriptan) has highlighted that the risk of first trimester birth defects was 4.2%, versus 3–5% in the general population.^18^ There are fewer data for other triptans. The current NICE CG150 headache guideline states that triptans can and should be considered for pregnant patients experiencing disabling headache attacks when other therapies have proven unhelpful and when patients have been counselled about their use in pregnancy. Triptans are generally considered compatible with breast feeding as less than 10% of the drug dose appears in breast milk; however, there are no large studies in this area.

#### Should we use triptans in patients with migraine aura?

Auras were previously thought to represent a vasoconstrictive episode and consequently there was concern about using triptans, which cause vasoconstriction, as this might in theory predispose to stroke. However, current understanding is that cortical spreading depression underlies aura, calling into question this theoretical contraindication.^19^ Studies of triptan use in familial hemiplegic aura report an excellent response to triptans and only minor side effects.^20^ Opinions vary among headache physicians, depending on the type of aura. Triptans are generally prescribed for migraine associated with non-motor aura. However, for hemiplegic migraine and also basilar migraine, there is less consensus, but the potential benefit of triptans may well outweigh their theoretical risks.

### Migraine prevention

Migraine preventative treatments aim to reduce the frequency and severity of attacks and may help reduce the frequency of analgesic use. It is usually unrealistic for patients to expect complete headache cessation and it can be useful to have a discussion with patients about realistic goals of treatment. Prophylactic treatment is typically considered if there are more than four migraine days per month. However, preventative therapy may also be given for less frequent but very disabling attacks.

Most medications used as preventatives were originally used for a different indication, and the evidence for individual agents varies. The agents used can be classified into antiepileptic drugs, beta blockers, antidepressants, serotonergic antagonists (methysergide is no longer manufactured), calcium channel antagonists, angiotensin modulators (ACE inhibitors and angiotensin-receptor inhibitors), nutrients and herbal products (Feverfew).

The current NICE guideline suggests first trying either topiramate or a beta blocker. If both are ineffective or contraindicated, then consider acupuncture, gabapentin, botulinum toxin or riboflavin.^8^ Since the publication of this guideline, there is new evidence that gabapentin is not effective, so we no longer use this.^21^ ^22^ The NICE guideline also concluded that there was insufficient evidence to support using amitriptyline and pizotifen, due to lack of robust clinical trials in this area.^8^ In reality, the choice of preventative depends in part on NICE guidelines, but is also influenced by patient comorbidities; thus, we also consider other agents as outlined in table 3.

**Table 3:** Migraine preventatives: an overview

Drug class	Drug	Side effects & contraindications	Target dose	Regimen
Beta blocker	Propranolol (alternatively atenolol or metoprolol)	Fatigue, depression, weight gain, bradycardia, impotence, orthostasis. Avoided in COPD/asthma, diabetes mellitus, peripheral vascular disease and those with bradyarrhythmias	80 mg BD (Atenolol 50–200 mg per day) (Metoprolol 100–200 mg per day)	Start 40 mg bd, titrate up to 160–320 mg daily
Serotonin antagonist	Pizotifen	Drowsiness, weight gain, dry mouth, urinary retention and manufacturers suggest avoiding in people with glaucoma, urinary retention, renal dysfunction and epilepsy	3 mg daily	Start at 0.5 mg OD, increase by 0.5 mg every 1–2 weeks
Antidepressant	Tricyclic Amitriptyline (alternatively dosulepin or nortriptyline)	Sedation, weight gain, dry mouth. Avoid amitriptyline in people with glaucoma, urinary retention, hypotension and significant cardiovascular comorbidity, including arrhythmias. Use with caution in people with epilepsy	50–75 mg daily	Start 10 mg ON with 10 mg ⇑ every 1–2 weeks
	SNRI Duloxetine (alternatively venlafaxine)	Constipation and diarrhoea, weight gain. Avoid in uncontrolled hypertension	60–90 mg	Start 30 mg, increase every week by 30 mg
Antiepileptic	Topiramate	Topiramate may have cognitive, anxiety and depression-provoking effects and may promote weight loss. Rarely renal calculi and glaucoma. Topiramate induces the metabolism of the combined contraceptive pill	100 mg	Start at 25 mg, ⇑25–50 mg every 1–2 weeks
	Valproate	Valproate can cause weight gain, tremor, alopecia and haematological dyscrasias. It can cause hyperammonaemia and is teratogenic. It should be avoided in liver disease (potentially hepatotoxic)	1000 mg	Start at 200 mg OD, ⇑ 200 mg every 2 weeks
Angiotensin based	Lisinopril	Lisinopril may cause fatigue, dry cough, angioedema, orthostasis or confusion. Hyperkalaemia or bone marrow dysfunction should be avoided	20–40 mg OD	Start at 10 mg OD, ⇑ to 20–40 mg
	Candesartan	Candesartan can cause vertigo and hypotension. It should therefore be avoided in individuals with these disorders	8 mg BD	Start 4 mg OD, titrate by 4 mg every week
Calcium channel blocker	Flunarizine	Weight gain, depression and extrapyramidal effects. May cause galactorrhoea in women concomitantly taking the combined contraceptive pill	5–10 mg	5 mg for a month, then 10 mg
Neutriceutical	Magnesium	In hypermagnesaemia, gastrointestinal (GI) effects, arrhythmia and coma are reported	600 mg daily	–
	Riboflavin	Physiologically limited absorption, limiting adverse effects	400 mg	–
	CoQ10	CoQ10 can cause usually mild GI effects, such as upset stomach	100 mg TDS	–

BD, twice daily; COPD, chronic obstructive pulmonary disease; OD, once daily; ON, once nightly; SNRI, serotonin norepinephrine reuptake inhibitor; TDS, three times a daily.

It is important to note that topiramate, flunarizine and beta blockers can exacerbate depression. Weight gain can be a concern with beta blockers, pizotifen, flunarizine, valproate and antidepressants. Beta blockers cannot be used in people with asthma (candesartan is a useful alternative with equivalent efficacy to propranolol). The tricyclic antidepressants, particularly amitriptyline, can be sedating though this can make it the drug of choice in people with sleeping difficulty. Topiramate can reduce the efficacy of the contraceptive pill. Also, many agents are potentially teratogenic and this should be mentioned to women of childbearing age.

The general rules of thumb are to start treatment at a low dose, gradually increasing to an initial target dose. If there is no effect and no significant side effects, the dose can be further increased for some drugs. We continue the medication for at least 3 months to evaluate efficacy. If effective (about a 50% improvement) the drug may be continued for 6 months, although there is some evidence of fewer rebound headaches if continued for 12 months.^23^

There is very limited evidence to support the use of SNRIs in migraine.

### Botulinum toxin for migraine

If three or more migraine preventatives have been trialled at effective dosages, the NICE guideline recommends doctors to consider botulinum toxin type A in chronic migraineurs (more than 15 headache days per month of which at last 8 are migrainous, for more than 3 months), provided there has been adequate management of medication overuse.

Botulinum toxin therapy should be stopped if it fails to provide the required response (a less than 30% reduction in headache days per month after two cycles), or if the headache has returned to an episodic phenotype.^10^ Earlier this year, longitudinal data from one of the largest botulinum toxin-treated migraine cohorts (refractory to preventative medications) demonstrated significantly more pain-free days and improved quality of life.^24^

### Pregnancy and migraine

Around 70% of migraineurs improve during pregnancy (especially second and third trimesters). If migraines are troublesome during the pregnancy, the choice of abortives should be discussed (see The triptans section). NSAIDs can be used in early pregnancy, but are absolutely contraindicated in the third trimester. Aspirin is a particular concern due to the possibility of developing Reye's syndrome in the neonate. Of the abortives, paracetamol is probably the safest.

During pregnancy, we tend to review patients with severe migraine more frequently to monitor progress and to discuss treatment options. There are understandably few data on which agents are safe in pregnancy and most drugs are unlicensed for use in pregnancy. Preventatives should be prescribed only if the potential benefits outweigh the potential risks. Low-dose (10–50 mg) amitriptyline is sometimes prescribed; however, there are reports of limb deformities following prenatal exposure to higher doses. Beta blockers may increase the risk of intrauterine growth retardation (25% risk with atenolol). Sodium valproate should be avoided. There is an excellent review of this topic available.^25^ Greater occipital nerve (GON) blocks may provide short to medium term analgesia. External neuromodulation using a single-pulse transcranial magnetic stimulation device was recently shown to be both safe and effective.^26^

### Menstrual migraine

Menstrual migraines have been attributed to falling oestrogen levels premenstrually and perimenstrually. ‘Perimenstrual mini’ prophylaxis (starting 2 days before the period and continuing during the period) can help, for example, frovatriptan 2.5 mg twice daily, zolmitriptan 2.5 mg three times a day, naproxen 500 mg twice daily or mefenamic acid 500 mg three times a day. Contraceptive-induced amenorrhoea can also be very effective, for example, with tricycling or continuous combined contraceptive pill.^27^ Oestrogen gels or patches can ameliorate the effect of falling serum hormone concentrations before and during the period (eg, 1.5 mg transcutaneous oestrogen days −2 to +5).

### GON block for migraine

**Figure 2:**
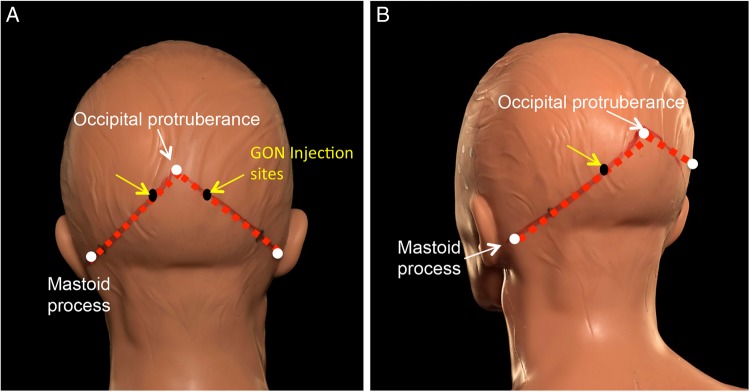
Injection sites for greater occipital nerve (GON) block. The injection sites (A and B; one to each GON) are identified as being at the proximal third of the distance between a hypothetical line from the occipital protruberance to the mastoid process. GON block side effects include a local alopecia, transient dizziness and worsening of the headache. The block can be repeated about every 3 months.

Neurologists working in the headache field, especially in the USA and latterly the UK, have used GON local anaesthetic blocks; these can provide analgesia for up to 2 months in chronic migraine sufferers. A recent double-blind randomised controlled trial of low-dose local anaesthetic block versus high-dose local anaesthetic block, however, challenged this notion of its sustained efficacy.^28^ A GON local anaesthetic block can provide a short-term rescue intervention for a patient in crisis. We sometimes use GON blocks to provide analgesia when starting prophylactic treatment in disabling refractory status migrainosus, to facilitate withdrawal of acute analgesics in those with medication-overuse headache and for patients with troublesome migraine in pregnancy. The exact composition of the nerve block can vary between centres. We tend to use 80 mg depomedrone (40 mg/mL) and 2 mL of 2% lignocaine, that is, total 4 mL per injection into each GON (figure 2). Some centres substitute the lignocaine for 1 mL of 0.5% bupivacaine. The efficacy and use of GON blockade in primary headache other than migraine, for example, cluster headache, is already better established.

### Migraine and the combined contraceptive pill?

Women with migraine and active aura have a twofold increased risk of ischaemic stroke.^29^ In women aged less than 45 years, smoking and the combined contraceptive pill further increase the stroke risk.^30^ NICE recommends avoiding combined hormonal contraceptives in women who have migraine with aura due to the increased risk of stroke. In these patients, we recommend the progesterone-only pill, contraceptive implant, depot or levonorgesterol intrauterine device.

### Treating headache in the emergency department

When managing acute, severe headache in the emergency department, our first concern is to identify red flags (see table 4). An accurate headache history is essential and if a true thunderclap or sinister headache is identified this will need investigating.

**Table 4:** Secondary headaches presenting to the emergency department

Cause	Clinical features
Vascular	
Subarachnoid haemorrhage	Nuchal rigidity, thunderclap headache, altered consciousness, nausea/vomiting, possibly focal neurological deficit (vasospasm, infarction)
Arterial dissection (carotid or vertebral artery)	Neck pain, focal neurological deficit if associated stroke
Venous sinus thrombosis	Headache (can be thunderclap), visual obscurations, papilloedema, focal signs, seizures. Increased risk with the combined contraceptive pill or other prothrombotic risk factors (eg, pregnancy)
Infective	
Meningitis	Fever/septic features, nuchal rigidity, nausea/vomiting, altered awareness, rash, photophobia
Encephalitis	As above, but also focal neurological deficit, confusion, seizures
Sinusitis/mastoiditis	Fever/septic features, sinus tenderness, altered hearing, nasal or aural discharge
Abscess	Fever/septic features, focal neurological signs, altered awareness, papilloedema
Ophthalmological	
Acute angle-closure glaucoma	Red eye, large oval pupil, unilateral visual disturbance
Inflammatory	
Giant cell arteritis	Age >50 years, unilateral visual loss, general malaise, weight loss, scalp tenderness, jaw claudication, raised erythrocyte sedimentation rate/serum C reactive protein)
Altered cerebrospinal fluid dynamics	
Idiopathic intracranial hypertension	Typically young women with high body mass index, visual obscurations/blurring, papilloedema and tinnitus
Colloid cyst of the third ventricle	Headache, gait disturbance, drop attacks
Low-pressure headache	Headache improved lying down, may be exacerbated by the valsalva manoeuvre
Space-occupying lesion with pressure effects	Headache (new, worsening, change in phenotype) may have associated lateralising features or seizure

When there is a positive diagnosis of severe migraine, its effective management can ensure high-quality patient care and also facilitates a timely discharge. We offer sumatriptan 6 mg, typically with an antiemetic such as metoclopramide or prochlorperazine. A recent systematic review advised against using morphine, tramadol, intramuscular diclofenac, haloperidol or dexamethasone.^31^ A period of intravenous rehydration may also help. It is also essential to make a future management plan to help prevent readmissions, which often are multiple in severe migraineurs. Clinicians can review the need for migraine prophylaxis and abortive therapy as an outpatient. A GON block can help patients in crisis before discharge.

## Medication-overuse headache

Medication-overuse headache is headache occurring on 15 or more days per month, developing as a consequence of regular overuse of acute or symptomatic headache medication (simple analgesics and NSAIDs on 15 days or more; or triptans, opioids and combination analgesics on 10 days or more days per month) for over 3 months. It occurs in patients with pre-existing primary headache who, in association with regular acute medication use, develop a new type of headache or a marked worsening of their pre-existing headache. Phenotypically, the headaches usually resemble migraine or tension-type headache.

Medication-overuse headache is a common, frequently debilitating, sequel to pre-existing headache. Headaches develop on withdrawal of analgesics, triggering more analgesic usage, leading to a self-perpetuating cycle. The problem is more severe with overuse of triptans and opiates than, for example, NSAIDs and simple analgesics, but can develop following overuse of any analgesic. A detailed look at a patient's list of over-the-counter medication can be quite revealing (table 5).

**Table 5:** Common over-the-counter migraine acute treatments

Symptomatic treatment	Contents
Anadin	Paracetamol
Anadin extra	aspirin, paracetamol and caffeine
Hedex extra	paracetamol and caffeine
Migraleve	paracetamol, codeine and buclizine
Panadol advance and actifast	Paracetamol
Panadol extra	paracetamol and caffeine
Panadol ultra	paracetamol and codeine
Paracodol	paracetamol and codeine
Paramol	paracetamol and dihydrocodeine
Solpadeine	ibuprofen and codeine
Solpadeine headache	paracetamol and caffeine
Solpadeine max	paracetamol, codeine, caffeine

**Figure 3:**
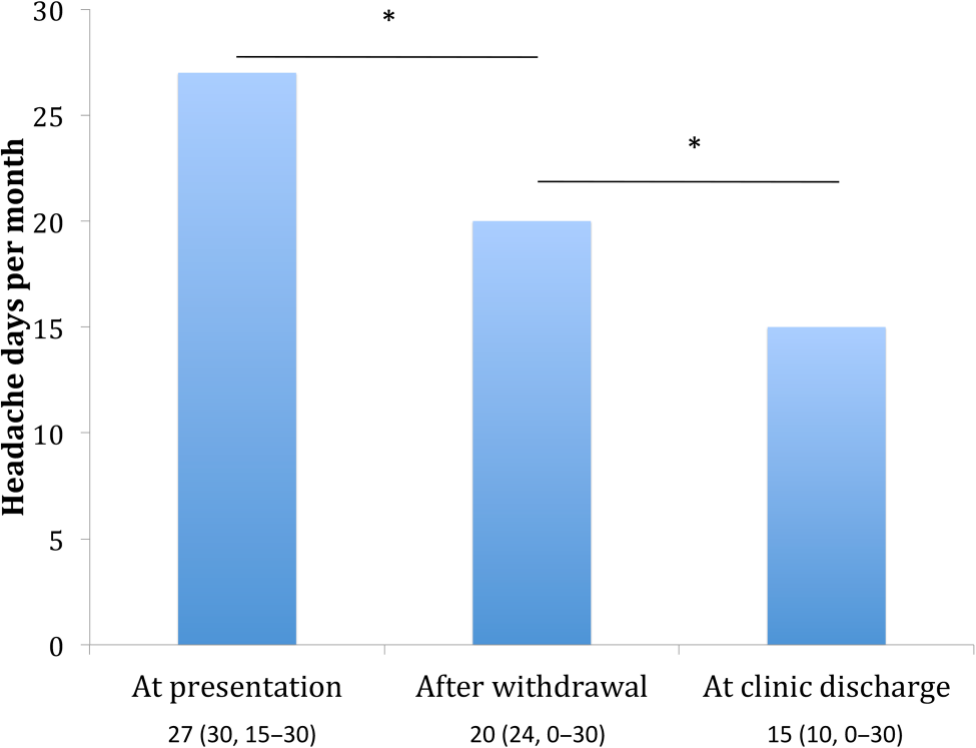
Headache frequency after medication withdrawal in medication-overuse headache. This Danish study of people with probable medication-overuse headache (175 patients) compared the frequency of headache before and after complete drug withdrawal and then after drug management in the clinic. Between the initial visit (before withdrawal) and discharge from clinic post-withdrawal, there was a mean reduction in headache frequency across the cohort of 46% (p<0.0001). The values represent mean reduction in headache days per month with median and range in brackets. *p<0.0001 (adapted from Zeeburg *et al*^44^).

Unfortunately, the process of withdrawing from analgesics is often challenging. Patients may develop not only a withdrawal or rebound headache but also nausea and other gastrointestinal disturbances (about a week for triptan withdrawal and up to 4 weeks for opiates and codeine-based medication). Before withdrawal, it is important that the patient is fully aware of the likely initial worsening of symptoms. The overused abortives should ideally be stopped for at least 1 month. It can be encouraging to the patient to explain the evidence that medication withdrawal alone can improve the headaches (see figure 3).

### How do we manage medication-overuse headache?

The aims of medication withdrawal are to reduce the headache frequency and severity and to optimise the efficacy of migraine preventatives. There is controversy as to whether migraine preventatives should be started while there is still ongoing medication overuse.

Most patients can withdraw their analgesics as an outpatient. They can opt either to stop the analgesics abruptly or alternatively to wean down by increasing, by 1 day each week, the number of medication-free days. Opiates and codeine-based medication may need to be gradually reduced, as the withdrawal symptoms can be severe. There is only sparse evidence supporting the use of alternative acute treatments during withdrawal. Naproxen 500 mg twice daily during the withdrawal can help as a short-term measure. We sometimes offer GON blocks to tide the patient through a period of drug withdrawal.

Some patients may need inpatient withdrawal, particularly those who also have substance abuse, additional unrelated medical morbidities or multiple high-dose analgesic overuse. Inpatient regimens may include intravenous hydration, dexamethasone, intravenous antiemetics and occasionally benzodiazepines or chlorpromazine. Some centres have access to and use intravenous aspirin (lysine acetylsalicylate) and/or intravenous dihydroergotamine with significant benefit.^32^

## Tension-type headache

Tension-type headache accounts for 45–80% of headache diagnoses in the general population. It is generally less severe than migraine, and patients may seek only reassurance. Many can manage with simple analgesia. People with chronic tension-type headache can use preventative treatments, for example, amitriptyline or topiramate.^33^ There is also evidence for non-pharmacological approaches, including acupuncture.^8^

## Cluster headache

Cluster headache is the most common of the trigeminal autonomic cephalalgias. Patients experience excruciating unilateral pain lasting 15–180 min at an attack frequency of 1–8 per day, usually at predictable times. The attacks occur in bouts of weeks to months and may repeat multiple times in a year with circannual periodicity. Pain is typically in an orbital, supraorbital and temporal distribution with associated autonomic features and agitation. Around 80% of cluster is episodic but in about 1 in 10 becomes chronic—headache attacks occurring for over 12 months without remission or with remission periods of less than 1 month.

### Management of cluster headache

Treatment strategies are either abortive (acute pain relief), transitional or prophylactic. The first-line abortive strategy is subcutaneous sumatriptan 6 mg (maximum two per day), with most people obtaining pain relief within 15 min (effective in 75%). If this cannot be used or tolerated, intranasal sumatriptan 20 mg or zolmitriptan 5 mg are alternatives but can take up to 30 min for their full effect. Home oxygen can be very useful at high flow, 100% concentration, via a non-rebreathe mask at 12–15 L/min for up to 20 min. In the UK, this is ordered via a Home Oxygen Order Form (HOOF-A). If carbon dioxide retention is a concern, we initially obtain an arterial blood gas sample and undertake formal respiratory assessment. If it is effective, then patients can use ambulatory oxygen to take to work and so on (in the UK, this requires a HOOF-B). These small cylinders contain around 30 min of oxygen, so patients need an ample supply.

Transitional treatments can help the patient get through a crisis period while starting preventative strategies. Prednisolone can be used, typically at 1 mg/kg up to 60 mg, for 5–7 days before tapering over 2 weeks. However, the efficacy rapidly diminishes during corticosteroid withdrawal and prolonged or multiple courses of corticosteroids carry significant side effects. GON blocks can also be very effective in reducing the attacks in the short term.^34^

Patients may need a preventative therapy to shorten the duration of the cluster bout and to help to control pain. Verapamil has proven efficacy in reducing the frequency of cluster headache after the first week, with a reduced median number of cluster headaches per day (1.65 on placebo vs 0.6 on verapamil).^35^ We perform ECG before starting verapamil, and again with each dose increase, looking for a prolonged electrocardiography P wave to R wave interval. A typical starting dose is 240 mg with 80 mg increments.^36^ Some centres use a faster titration although there are no safety studies to evaluate this regimen (see online supplementary table S1). Second-line agents include lithium carbonate (see online supplementary table S2) and topiramate. There is less evidence for gabapentin, melatonin and sodium valproate but we may occasionally use these if other agents fail.

### Future directions for cluster headache management

There is growing evidence for using neuromodulation (both invasive and non-invasive) in headache; this appears a promising avenue for future treatment, which we await with enthusiasm. Among the neuromodulation options, non-invasive vagus nerve stimulation (GammaCore) shows promising results for cluster headache and migraine. There have also been very encouraging results using the invasive sphenopalatine ganglion stimulator to reduce pain in patients with intractable chronic cluster headache.^37^ We shall cover this in more detail in a linked article.

## Future treatments

Future headache treatments have several key themes. The increased scrutiny of widely used antiepileptic drugs as possible migraine treatments, the evaluation of new modes of triptan delivery and the use of drug from altogether novel classes.

### Calcitonin gene-related peptide (CGRP) therapy

A promising novel strategy focuses on calcitonin gene-related peptide (CGRP). This is a potent vasodilator, a trigger for migraine episodes and is raised during migraine and cluster attacks. Early CGRP antagonist trials started well but have been halted due to adverse liver reactions. However, early data from CGRP antibody therapy trials are encouraging.^38^ CGRP antibodies used as abortive agents are attractive compared with triptans as they do not cause vasoconstriction, and so potentially can help a larger population of patients.

### New triptan formulations and delivery methods

There are several new methods to deliver triptan therapies.

- The Optinose apparatus (Avanir) delivers sumatriptan powder into the higher nasal mucosa through blowing into the device; initial evidence suggests that this is rapidly effective. It may soon be available in the USA.^39^
- Needle-free subcutaneous sumatriptan, delivered through a high-pressure canister into the skin (Sumavel), may be useful in needle-phobic patients.^40^
- The Zecuity patch (NuPathe) is an FDA-approved transdermal sumatriptan patch that permits electronically controlled delivery.
- A new zolmitriptan oral dispersible film (Applied Pharma Research, Labtec, MonoSolRx) has been approved for use in most of Europe.
- New serotonin agonists, such as lasmiditan, have greater selectivity for the desired 5-HT_1F_ receptors. These may also have a role due to their ability to minimise risks of significant vasospasm through unwanted binding of other serotonin receptors. Lasmiditan was effective in trials of acute therapy of migraine.^41^

### Antiepileptic drugs

Antiepileptic drugs, not historically appreciated for their benefit in managing migraine, have generated heightened interest recently—particularly levetiracetam and lacosamide—with the recognition of the role of cortical hyperexcitability in migraine development. We need further studies to evaluate their efficacy.^42^

### Other agents

As knowledge of the pathogenic mechanisms involved in headache increases, new agents are developed specifically to target headache pain. Melatonin receptor agonists, for example, ramelteon, were developed following evidence of pineal dysfunction in headache. Trials are currently underway in this area (NCT00739024). Tonabersat is currently being evaluated as a drug to inhibit spreading cortical depression.^43^

## Conclusions

The effective management of headache disorders remains a moving field and a potential challenge to the neurologist. The frequency of headache and its burden of disability make it essential for neurologists to manage headache in an informed way. Therapeutic decisions are hampered by minimal evidence to support the use of many therapies and hence we a need large, robust clinical trials. However, emerging therapies specifically targeting headache pain pathways are likely to improve significantly the management options and advance the field.

A practical approach is important—using existing evidence and guidelines—to enable effective headache management. It is an area where the clinician can have a huge and very rewarding impact upon patient care.

**Key points**

- Headache is a common cause of disability; an accurate diagnosis is essential, though is often delayed or missed.
- Medication-overuse headache frequently coexists and should be actively sought and discussed with patients with headache.
- National Institute of Health and Care Excellence (NICE) recommends acute migraine abortive treatment with a triptan together with paracetamol (or a non-steroidal anti-inflammatory drug) plus an antiemetic; lack of efficacy with one triptan does not mean that others will not work.
- NICE guidelines for migraine prophylaxis suggest using topiramate or a beta blocker; comorbidities or lack of efficacy often means that other agents are also prescribed.
- Start migraine prophylactic agents at low dose, build the dose up progressively and maintain a therapeutic dose for 3 months before judging its efficacy.

## Supplementary Material

Web supplement
