# Supplementary material for: Headache management: pharmacological approaches
Source: Pract Neurol. 2015 Jul 3;15(6):411–23. doi: 10.1136/practneurol-2015-001167 (PMC4680181; doi:10.1136/practneurol-2015-001167)
Supplement: Web supplement [file practneurol-2015-001167-s1.pdf]

Supplemental Tables

Table 1 Regime for titrating verapamil for Cluster Headache (CH)

The table below outlines the method we use for escalating verapamil, as a cluster headache preventative.

	**Morning**	**Midday**	**Evening**
For 2 weeks take*	120mg		120mg
For 2 weeks take	120mg	120mg	120mg
For 2 weeks take	120mg	120mg	240mg
For 2 weeks take	240mg	120mg	240mg
For 2 weeks take	240mg	240mg	240mg
For 2 weeks take	240mg	240mg	360mg
Thereafter take	360mg	240mg	360mg

**It is important to ensure that an ECG is performed prior to initiating and every increase in verapamil dosage.*

Table 2 Regime for titrating lithium for Cluster Headache (CH)
The table below outlines the method we use for escalating lithium, as a cluster headache preventative.

	**Treatment**	**Investigations**	**Action**
Baseline		Renal and thyroid function	Only start lithium if these are normal
**Weeks 1-2**			
Day 1	Start lithium 300mgs twice daily and stay on this dose for 2 weeks		
Day 7		Blood test for lithium level	
Day 14	Alter lithium dose as outlines in "action" section		• If headaches resolved or significant side effects develop, then re-evaluate.<br>• If lithium level < 0.8 then increase dose by 100mgs twice daily (i.e. if on 300mgs twice daily then increase dose to 400mgs twice daily).<br>• If lithium level 0.8-1.0, then do not increase dose unless advised by your medical team.<br>• If lithium level >1.0 then will probably need to reduce the lithium dose.
**Weeks 3-4**	Stay on the lithium dose started on day 14 for two weeks		
Day 21		Blood test for lithium level	
Day 28	Alter lithium dose as outlines in "action" section		• If headaches resolved or significant side effects develop, then then re-evaluate.<br>• If lithium level < 0.8 then increase dose by 100mgs twice daily (i.e. if on 300mgs twice daily then increase dose to 400mgs twice daily).<br>• If lithium level 0.8-1.0, then do not increase dose unless advised by your medical team.<br>• If lithium level >1.0 then will probably need to reduce the lithium dose.
Weeks 5-6	Continue this cycle as required		
